# Long-term exposure to environmental concentration of dinotefuran disrupts ecdysis and sex ratio by dysregulating related gene expressions in *Chironomus kiinensis*

**DOI:** 10.3389/fendo.2024.1459329

**Published:** 2024-09-05

**Authors:** Fenghua Wei, Shuangxin Wu, Long Li

**Affiliations:** ^1^ School of Chemistry and Environment, Jiaying University, Meizhou, China; ^2^ School of Physics and Electrical Engineering, Jiaying University, Meizhou, China

**Keywords:** neonicotinoid insecticides, environmental concentration, endocrine disruption, Chironomidae, sex ratio

## Abstract

**Introduction:**

Currently, although there have been a few reports on the endocrine-disrupting effects of neonicotinoids, the effect on Chironomidae during long-term exposure remains unknown.

**Methods:**

Ecdysis and sex ratio, along with ecdysone-relevant gene expressions of representative neonicotinoid dinotefuran on *Chironomus kiinensis* were investigated at different environmental concentrations by long-term exposure.

**Results:**

A low dose of dinotefuran delayed pupation and emergence via inhibiting ecdysis. Sex ratios of adults shifted toward male-dominated populations with the concentration of dinotefuran increasing. The corresponding transcriptions of ecdysis genes *ecr, usp, E74*, and *hsp70* were significantly downregulated in the midge. For estrogen effects, the *vtg* gene expression was upregulated, but there was no significant change for the *err* gene.

**Discussion:**

These results would improve our understanding of the endocrine-disrupting mechanisms of neonicotinoid insecticides to Chironomidae and provide data support for assessing their potential environmental risks.

## Graphical Abstract

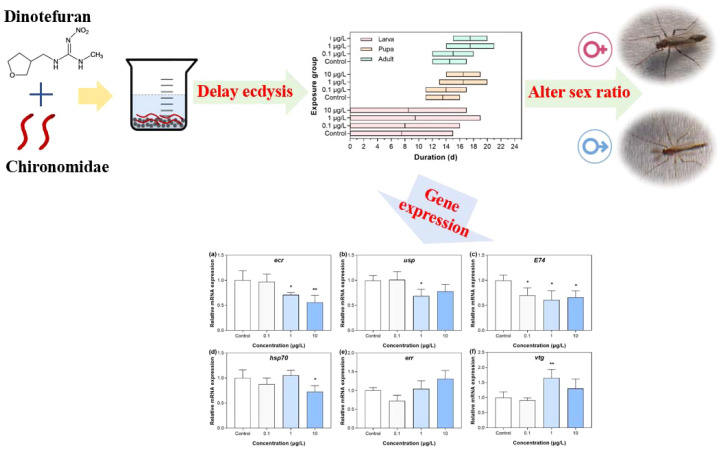

## Highlights

- Low dose of dinotefuran delayed growth and development via inhibiting ecdysis.
- Dinotefuran shifted sex ratios toward male-dominated populations.
- Dinotefuran downregulated gene expressions related to ecdysis hormones.

## 1 Introduction

Neonicotinoids are one of the most commonly used pesticides worldwide in agriculture, due to their broad spectrum, high efficiency, and low toxicity (1). Thus, neonicotinoids’ widespread use, combined with moderate persistence, leads to their ubiquity in the environment (2). Neonicotinoids are designed to target the nicotinic acetylcholine receptors (nAChRs) in insect selectively and disturb the central nervous system, leading to insect paralysis and death (3). Because of the unique mechanism of action, neonicotinoids cause less harm to non-target organisms and the environment compared with the highly toxic organophosphorus pesticides (4).

However, rising evidence revealed that neonicotinoids exhibited a number of additional toxicities (e.g., mitochondrial dysfunction, endocrine disruption, reproductive toxicity, and immunotoxicity) to non-target organisms, including vertebrates and even human beings (5, 6). The exploration of their additional toxic mechanism has become an emerging focus of public attention. Among them, the endocrine-disrupting effects of neonicotinoids have been confirmed by several studies. Computer molecular docking simulations and *in vivo* experiments showed that imidacloprid could bind to the thyroid receptor (TR) in mice by competing with T3, a thyroid hormone (7). Flupyradifurone, a new nicotinoid insecticide, induced sex-based dysregulation of the hypothalamic–pituitary–thyroid axis of rats, exhibiting the potential agonistic or antagonistic effect of flupyradifurone sex-dependent metabolites on liver thyroid hormone receptors (8). Imidacloprid also interfered with the pituitary–thyroid axis of red munia (9). Long-term exposure to acetamiprid altered hormone production and the related gene expression of the hypothalamic–pituitary–gonad (HPG) axis in a sex-dependent manner and caused feminization and reproductive dysfunction in zebrafish and their offspring (10). Imidacloprid disrupted larval molting regulation, causing developmental delay in honey bee (11). Although progress has been made in understanding the endocrine-disrupting effects of neonicotinoids on many non-target species, especially vertebrates, this toxicity remains poorly understood in aquatic insects that belong to Chironomidae.

Chironomid larvae play important ecological functions as they have a large biomass in freshwater ecosystems and are natural food for many aquatic organisms (12). In addition, Chironomidae are more sensitive to neonicotinoids than other aquatic invertebrates (13). Chironomids are metamorphosed species with four life stages (egg, larva, pupa, and adult). For the larva, it undergoes three ecdysis, then molts into a pupa, and finally molts into an adult. Therefore, the molting activity is crucial in the whole life process, which is regulated by ecdysone and juvenile hormones. Previous studies have shown that neonicotinoids arrested pupal ecdysis in *Lepidoptera* (14) and disrupted larval molting regulation, causing developmental delay in honey bee (11). Whether the molting activity of chironomids that are more sensitive to neonicotinoids is also affected by the insecticide, the question remains, which genes regulate their effects? Therefore, it is urgent to uncover the ecdysis effect of neonicotinoids on chironomids and its regulatory mechanism at different environmental concentrations for long-term exposure.

Dinotefuran, as a third-generation neonicotinoid insecticide, has accounted for more than a quarter of the global pesticide used due to its excellent properties, such as higher insecticidal activity, quicker uptake, smaller resistance, and more safety for the environment and humans (15–17). A few studies reported that the detection concentration was 0.012–0.8 μg/L in environmental waters (18–20). Raby et al. (21) reported the chronic toxicity of dinotefuran to *Chironomus dilutus*, showing that the 14-day lethal concentration of 50% (LC_50_) and emergence concentration of 50% (EC_50_) were 15.1 (13.4–16.8) μg/L and 8.15 (7.35–8.95) μg/L, respectively. However, the toxic mechanisms of the endocrine-disrupting effects of dinotefuran are poorly studied to date.

In the present study, the endocrine-disrupting effects of representative neonicotinoid dinotefuran on *Chironomus kiinensis* were investigated at different environmental concentrations by long-term exposure. Ecdysis and sex ratio, along with relevant gene expressions, were measured. These results would improve our understanding of the endocrine-disrupting mechanisms of neonicotinoid insecticides to Chironomidae and provide data support for assessing their potential environmental risks.

## 2 Materials and methods

### 2.1 Materials

Dinotefuran, thiamethoxam-*d*_3_ (internal standard), and imidacloprid-*d*_4_ (surrogate standard) were purchased from Dr. Ehrenstorfer GmbH (Augsburg, Germany) with purity >98%. *C. kiinensis* were cultured in Jiaying University according to the standard protocol of USEPA2000 proposed by the U.S. Environmental Protection Agency.

### 2.2 Toxicological assay

Test water was freshly prepared with 0.1, 1, and 10 μg/L of dinotefuran (DIN_1, DIN_2, and DIN_3) into reconstituted moderately hard water. Negative control and solvent (0.1% DMSO) controls were tested in the meantime. A 0.5-cm layer of quartz sand and 200 mL of the testing solution were introduced into each 500-mL beaker. Twenty newly hatched midge larvae (within 24 h) were randomly added into each beaker with three replicates per treatment group or control group. The organisms were fed ground fish food every 2 days per beaker and the test solution was changed every 5 days. Water quality parameters (i.e., conductivity, pH, temperature, and dissolved oxygen) in test solution were monitored every day and ammonia nitrogen was monitored on days 0, 5, and 10. Three experimental groups were tested in the chronic bioassays. For the first group, mortality, pupation, emergence time, and sex ratio were recorded daily from the first emergence at 11 days and ended until the last chironomid completed emergence (approximately 21 days). For the second group, the survival larvae at 11 days were evaluated for adenosine triphosphate (ATP) levels. The third group at 11 days was evaluated for the levels of gene expression.

### 2.3 Quantification of dinotefuran

Exposure samples were collected from test containers at 5 and 10 days of exposure in three replicates and analyzed for dinotefuran actual concentrations using HPLC-MS/MS following a previously developed method from Wei et al. (22). In brief, the sample was extracted using solid-phase extraction (SPE) cartridges packed with 200 mg of HLB absorbent. Before loading the sample, the SPE cartridge was conditioned with 3 mL of methanol and 10 mL of water, sequentially. Samples were then passed through the cartridges at a flow rate of 3–5 mL/min and eluted out of the cartridges with 10 mL of methanol. Eluants were evaporated to near dryness under a gentle flow of nitrogen and re-dissolved in 500 μL of acetonitrile. The HPLC-MS/MS analysis was conducted on a Shimadzu DGU-30A HPLC coupled with an AB SCIEX TRIPLE QUAD™ 5500 tandem MS system. The analytes were separated on an Agilent Zorbax Eclipse Plus C18 column (100 × 2.1 mm i.d., 1.8 μm) at 40°C. The mobile phase was a mixture of water containing 0.1% formic acid (A) and acetonitrile (B) and flow rate was 0.3 mL/min. The gradient elution condition was as follows: 0 min, 37% B; 1.2 min, 37% B; 3 min, 70% B; 3.5 min, 70% B; 3.6 min, 37% B; and 5.1 min, 37% B. The injection volume was 2 μL. The MS monitoring was performed using an electrospray ionization (ESI) source in positive mode and multiple reaction monitoring (MRM). The MS/MS conditions were as follows: source temperature, 550°C; curtain gas (CUR), 40 psi; collision gas (CAD), 7 psi; ion source gas 1 (GS1), 55 psi; ion source gas 2 (GS2), 55 psi; ion spray voltage (IS), 5,500 V; entrance potential (EP), 10 V; and collision cell exit potential (CXP), 16 V. Other MS parameters for dinotefuran qualification are listed in **Supplementary Table S1**. Quantification of dinotefuran was achieved using an internal standard calibration method, and the calibration curve for dinotefuran was linear over a range of 0.1–50 μg L^−1^.

### 2.4 Measurements of ATP level

For the second group, ATP was measured using a commercial ATP assay kit (Nanjing Jiancheng, China) according to the manufacturer’s protocols. Creatine kinase catalyzes ATP and creatine to produce creatine phosphate, which was detected by phosphomolybdate colorimetry. After 11 days of exposure, survival larvae in control and three treatment groups (DIN_1, DIN_2, and DIN_3) were homogenized in 1 mL of cool ultrapure water for 3 min. Part of the homogenate was centrifuged at 3,500 rpm at 4°C for 10 min and the supernatant was taken to measure the protein concentration. The other homogenates were then boiled in a boiling water bath for 10 min, mixed, and extracted for 1 min, and the supernatant was centrifuged at 3,500 rpm at 4°C for 10 min. Then, the supernatant was used for measuring the ATP level at 636 nm by the microplate reader.

### 2.5 Measurements of gene expression levels

For the third group, surviving larvae from DIN_1, DIN_2, and DIN_3 were immediately frozen with liquid nitrogen before use. Total RNA was isolated using an RNeasy Mini Kit (Qiagen, Hilden, Germany) according to the manufacturer’s protocol. Expressions of six genes (**Table 1**) were quantified using a real-time quantitative polymerase chain reaction (RT-qPCR). Primers were either chosen from previous studies or designed by NCBI/Primer-BLAST. *β*-actin was chosen as an internal control. The RNA samples were reversely transcribed into cDNA by using a Bestar™ qPCR-RT Kit (DBI-2220, German). RT-qPCR was performed in an ABI 7500 fluorescence quantitative PCR instrument (ThermoFisher, USA). The fold changes of the target genes were calculated using the 2^−ΔΔCT^ method (25).

**Table 1:** Primers used for the amplification of specific genes.

Gene	Sequence (5′–3′)	Fragment size (bp)	Reference
*β*-actin	F: ATGAATTGCCCGATGGACAA R: ACCGCATGATTCCATACCCA	101	–
*ecr*	F: AGGATCAAGAGCACGAGGCA R: CCCTTTTGCGAATTCCACAA	86	–
*usp*	F: CCGCCCAATCATCC R: CTGTGCGTTTGAAGAATCC	121	(23)
*E74*	F: TCTTACTGAAACTTCTTCAAGATCG R: GCTTTGAGACAGCTTTGGAATCG	111	(23)
*hsp70*	F: AATGACTCGCAACGTCAAGC R: AGTGCTGCTGCAGTTGGTTC	92	–
*err*	F: TAAGCGCAGGAGGAAAGCAT R: GCCTTCCCCCTCGAACTCTA	104	–
*vtg*	F: GATTGTTCCATGTGCAG R: TTTGAGTATGGTGGAGAATC	215	(24)

### 2.6 Statistical analysis

Differences among the treatments were analyzed with one-way ANOVA by SPSS 17.0 software (SPSS Inc., Chicago, IL, USA). Normality of each data set was assessed using the Kolmogorov–Smirnov one-sample test. Significant differences were evaluated by one-way ANOVA with LSD’s *post-hoc* test where data met the assumptions of normality and homogeneity of variance. *p-*value < 0.05 was considered statistically significant.

## 3 Results

### 3.1 Dinotefuran delayed the ecdysis activity of chironomids

**Figure 1:**
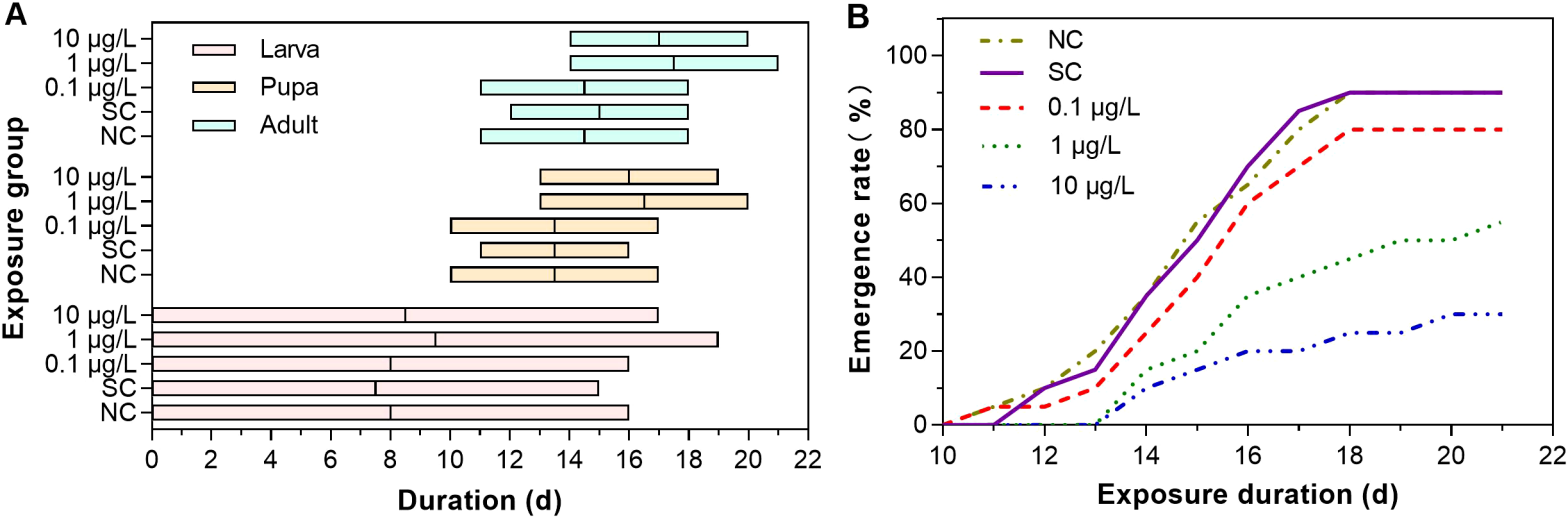
Duration of ecdysis of midges **(A)** and total emergence **(B)** of *C. kiinensis* adults after exposure to different concentrations of dinotefuran. NC, negative control; SC, solvent control.

Dinotefuran concentrations in exposure solutions varied little during the experiment duration (**Supplementary Table S2**). For the first group, durations of different life stages of midges were different among the dinotefuran exposure and control groups (**Figure 1A** and **Supplementary Table S3**). For the larva stage from first instar to fourth instar, life duration in the negative control, solvent control, and DIN_1, DIN_2, and DIN_3 groups was 16, 15, 16, 19, and 17 days, respectively, showing an extended trend compared to the control group, which indicated that the molt of larvae was delayed by dinotefuran in each instar. For the pupa stage, the time of the first pupa was 10, 11, 10, 13, and 13 days for the negative control, solvent control, and DIN_1, DIN_2, and DIN_3 groups, respectively, indicating that pupation of DIN_2 and DIN_3 groups was delayed. For the adult stage, the emergence of the first pupa was also delayed at DIN_2 and DIN_3 groups (14 and 14 days) relative to the negative control (11 days) and solvent control (12 days) except for DIN_1 (11 days). Meanwhile, the time of the last emergence was delayed at DIN_2 and DIN_3 groups (21 and 20 days) relative to the controls (18 days). For **Figure 1B** and **Supplementary Table S4**, the emergence rate decreased with the increase of exposure concentration of dinotefuran. These results suggested that molting of larva and pupa was inhibited by dinotefuran.

### 3.2 Dinotefuran altered sex ratios of adults

**Figure 2:**
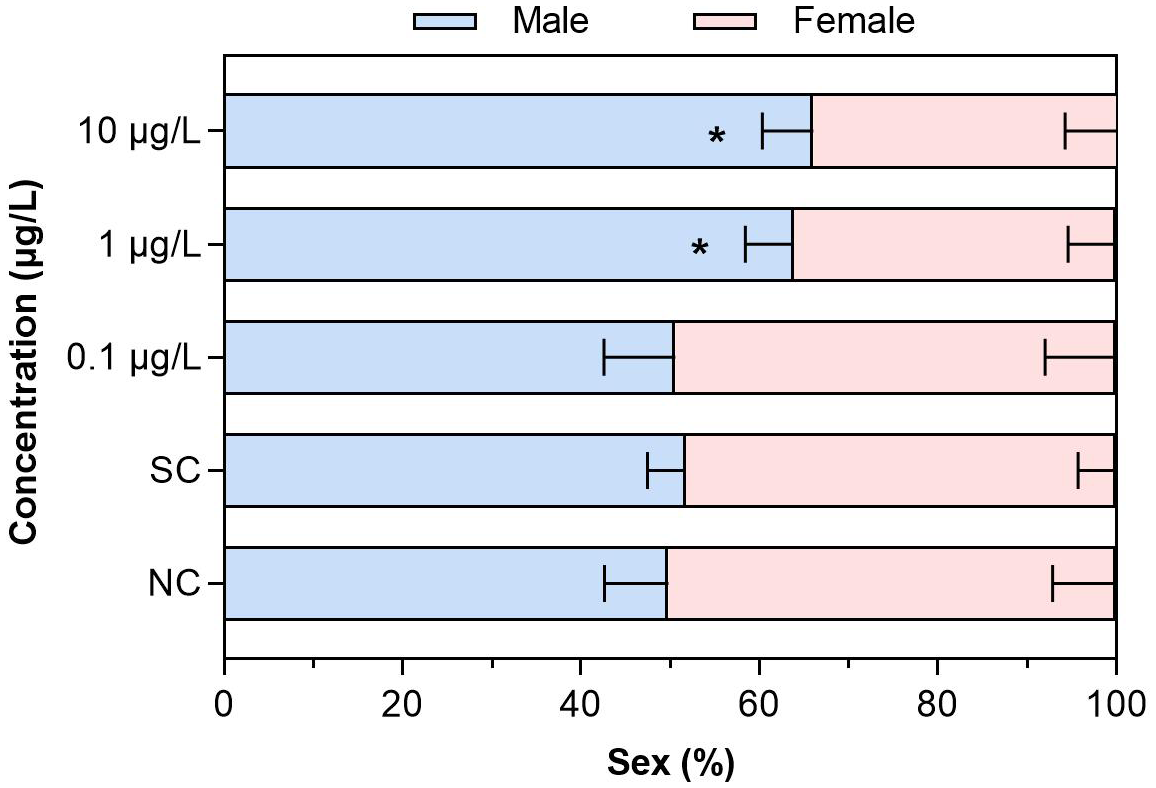
The sex (%) of *C. kiinensis* adults after chronic exposure to different concentrations of dinotefuran. Significance compared with solvent control was tested by ANOVA (*n* = 3, *p < 0.05). NC, negative control; SC, solvent control.

After the larva of *C. kiinensis* was chronically exposed to different concentrations of dinotefuran, dinotefuran revealed its effects on the sex of emerged adults for the first group (**Figure 2** and **Supplementary Table S5**). In the negative control and solvent control groups, 49.8% ± 7.1% and 51.7% ± 4.3% of the larvae were male, respectively. For the dinotefuran exposure groups DIN_1 to DIN_3, 50.6% ± 8.0%, 63.8% ± 5.4%, and 66.0% ± 5.7% of the larvae were male, respectively. Chronic exposures to dinotefuran elicited trends of significant sex ratio shifts toward male-dominant populations with concentrations increasing relative to the control.

### 3.3 Dinotefuran reduced ATP levels

**Figure 3:**
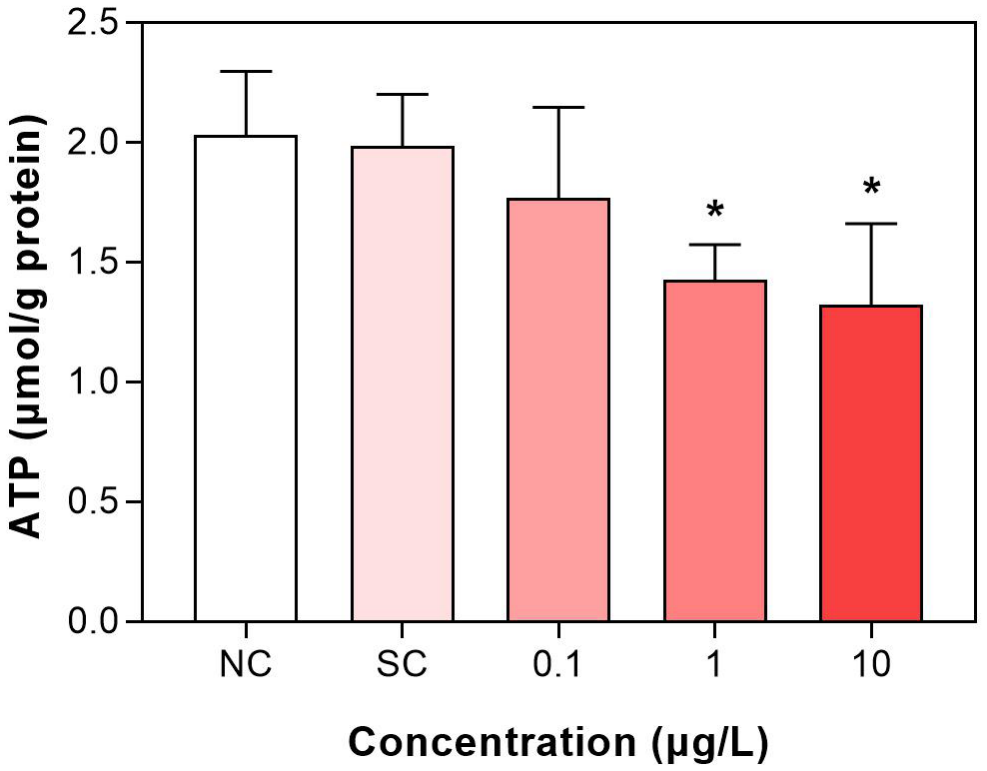
Adenosine triphosphate (ATP) level of *C. kiinensis* after 11 days of exposure to different concentrations of dinotefuran. Significance compared with solvent control was tested by ANOVA (*n* = 3, *p < 0.05). NC, negative control; SC, solvent control.

The levels of ATP in the second group were decreased after exposure to dinotefuran (**Figure 3** and **Supplementary Table S6**). At 0.1 μg/L (DIN_1) of exposure concentration, the ATP was decreased but without significant difference. At higher concentrations, 1 and 10 μg/L (DIN_2 and DIN_3), significant reduction (*p* < 0.05) was observed.

### 3.4 Dinotefuran dysregulated the levels of gene expressions related to ecdysone

**Figure 4:**
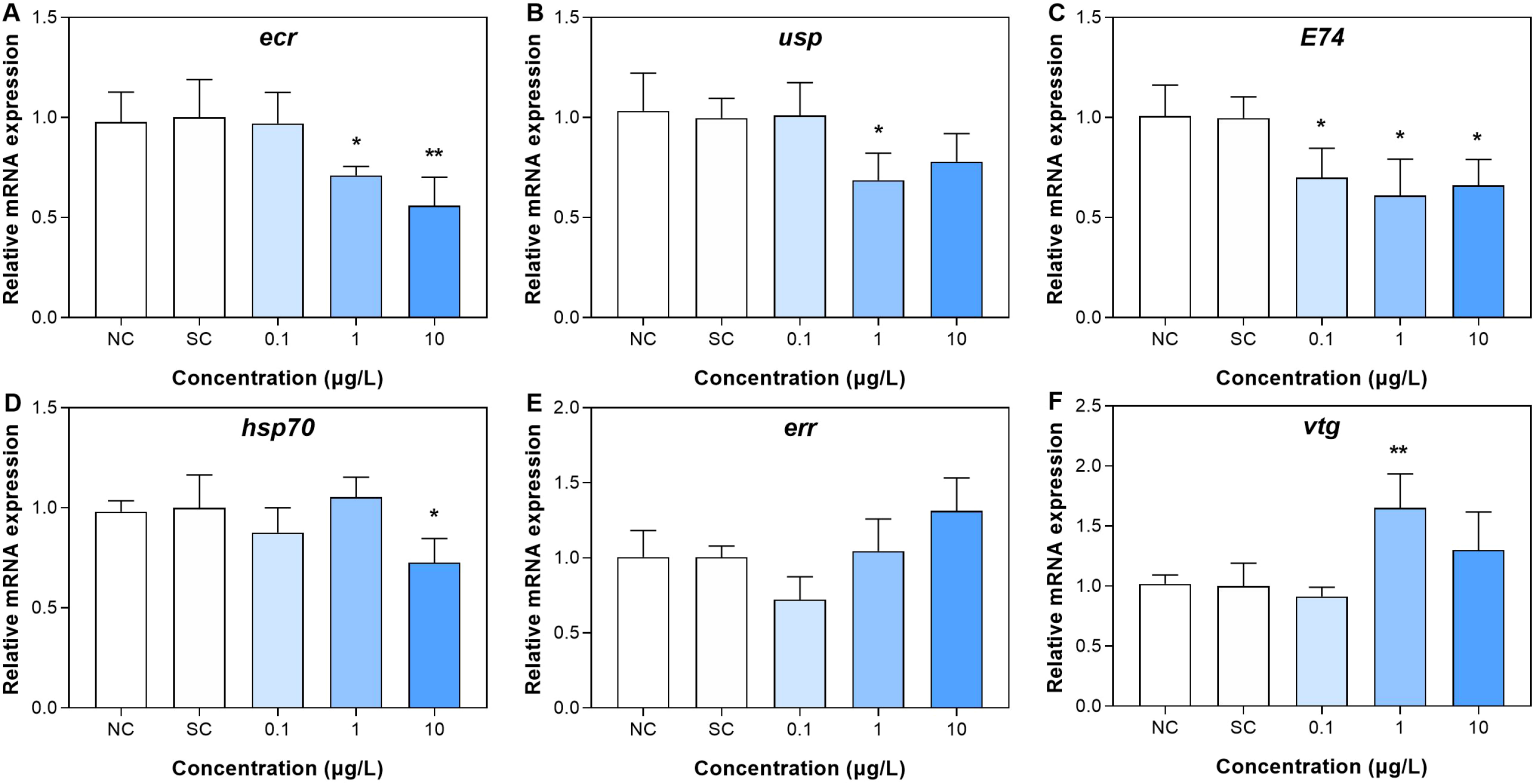
Gene expressions related to ecdysone **(A–D)** and estrogen **(E, F)** effect in larva after 11 days of exposure to dinotefuran. Data are expressed as mean ± standard error (*n* = 3). Significance compared with solvent control was tested by ANOVA (*n* = 3, *p < 0.05, **p < 0.01). NC, negative control; SC, solvent control.

For the third group, the gene expressions in the larvae were showed in **Figure 4** and **Supplementary Table S7**. In terms of the molting, the gene expressions of *ecr* (ecdysone receptor) at DIN_2 and DIN_3 groups (1 and 10 μg/L) were significantly downregulated relative to the control group (**Figure 4A**), showing that with the increase of exposure concentration, the greater the downregulation on the gene expression. For *usp* (ultraspiracle protein, related to transcriptional effects of ecdysone) gene, its expression was significantly decreased only in the middle group (DIN_2, 1 μg/L) (**Figure 4B**). For *E74* (an inducible factor related to ecdysone) gene, its expression was significantly decreased in the three exposure groups (DIN_1 to DIN_3) compared with the control group (**Figure 4C**). The gene expression of *hsp* (heat shock protein, playing a key role in the synthesis and maturation of steroid hormone receptors) was only significantly decreased in the highest exposure group (DIN_3, 10 μg/L, **Figure 4D**). For estrogen effects, gene expression of *err* (estrogen-related receptor) was not significantly altered in all exposure groups of dinotefuran, while the expression of *vtg* (vitellogenin, related to the estrogen effect) gene was upregulated in the DIN_2 group (1 μg/L) compared to the control (**Figures 4E, F**).

## 4 Discussion

Neonicotinoid insecticides are widely used as alternatives of older classes of pesticides because they are less persistent in the environment and less toxic to mammals (including humans). It is well known that neonicotinoids damage the nervous system by binding to nAChRs, eventually leading to paralysis and even death of target insects (26). However, more and more studies found that besides targeting nAChRs, neonicotinoids could also induce other effects, such as interfering with energy metabolism in Chironomidae (22) and *Xenopus laevis* (27), leading to endocrine-disrupting effects in zebrafish (10). In particular, in terms of endocrine-disrupting effects, the mechanism of toxicity of neonicotinoid insecticides in Chironomidae is unclear.

### 4.1 Dinotefuran delayed the ecdysis activity of chironomids by regulating ecdysone-related gene expressions and reducing the ATP levels

In this study, the endocrine-disrupting effects of the representative neonicotinoid pesticide, dinotefuran, were investigated on Chironomidae. Our results found that dinotefuran delayed the ecdysis behavior of midges, especially during pupation and emergence at 1 and 10 μg/L. A few studies reported detected concentrations of dinotefuran in various water bodies. For example, Putri et al. (19) presented that the highest concentration of dinotefuran was 0.023 μg/L in estuaries and mangrove areas in tropical environmental waters of Indonesia. Xiong et al. (18) detected dinotefuran from a paddy field to receiving waters in Poyang Lake basin of China, with a mean concentration of 0.200 ± 0.296 μg/L and a maximum concentration of 0.802 ± 0.139 μg/L. Dinotefuran was also detected with a concentration of 0.0127–0.0755 μg/L in rivers near maize field in Ontario, Canada (20) and 0.0016–0.134 μg/L in streams across the United States (28). Thompson et al. (29) investigated dinotefuran in well tap water in eastern Iowa with a maximum concentration of 0.0039 μg/L. From the above reports, the range of dinotefuran was approximately 0.002–0.8 μg/L. The present study showed that 1 μg/L of dinotefuran delayed the ecdysis activity of chironomids, which was close to the highest environmental concentrations. Our results indicated that the effects of long-term exposure to environmental concentrations of dinotefuran on the growth and development of chironomids cannot be ignored.

For metamorphosed species, ecdysis is an important part of their life development. Li et al. (11) reported that imidacloprid caused honey bee larval developmental retardation by disrupting larval molting regulation. Krishnan et al. (14) revealed that neonicotinoids can cause arrested pupal ecdysis and adult eclosion in *Lepidoptera*. Ecdysis requires a large amount of energy supply, so the delay of ecdysis by neonicotinoids may be related to the energy interference effect of neonicotinoids. In this study, the levels of ATP were significantly decreased after exposure to dinotefuran at 1 and 10 μg/L (DIN_2 and DIN_3). A number of studies have reported that neonicotinoids could induce mitochondrial dysfunction. For example, Zhu et al. (27) revealed that chronic exposure to environment-related concentrations (5 μg/L) of dinotefuran led to mitochondria fusion and disturbed the mitochondrial respiratory chain of *X. laevis*, reducing ATP levels. Our previous study also showed that after exposure to the neonicotinoid insecticide imidacloprid, mitochondria cristae of Chironomidae disappeared and the morphology of mitochondria was altered using ultrastructural analysis; subsequently, the ATP levels were also reduced (22). Li et al. (11) also revealed that disruption of nutrient energy metabolism induced by imidacloprid caused developmental delay in honey bee.

The metamorphosis development of chironomids is mainly regulated by the combination of ecdysone and juvenile hormone, which control morphological events during ecdysis. Ecdysteroids promote pupation, while juvenile hormones maintain larval characteristics and prevent metamorphosis by countering the effects of 20-ecdysterone (20E) (30, 31). Dinotefuran inhibited the molting of chironomids, thus delaying their growth and development. It is possible that dinotefuran affected the secretion of ecdysone and juvenile hormone. The transcriptional effects of insect ecdysteroids require the action of two nuclear receptor superfamilies, the ecdysone receptor (EcR) and the ultraspiracle (USP). Activation of EcR/USP heterodimers initiates a series of expressions of ecdysone effect genes, leading to the disappearance of larval organs, as well as the differentiation of adult tissues (24). Our results showed that the gene expressions of *ecr* and *usp* were downregulated in groups treated with 1 and 10 μg/L of dinotefuran, manifesting that the transcriptions of ecdysteroids were inhibited, which interfered with normal molting activities. Li et al. (2023) reported that imidacloprid reduced 20E titer and inhibited Br-c expression, thereby blocking molting and causing stunting of honey bee larvae. Meanwhile, the developmental regulatory gene juvenile hormone acid methyl transferase (*jhamt*) was downregulated. In this study, environmental concentration of dinotefuran also downregulated the gene expression of *E74*. Among the ecdysone response genes, *E74* transcription factor is an early gene of ecdysone induction (24). It plays a key role in the time of insect metamorphosis, and the expression pattern of this gene is associated with pupation (24). In addition, the hsp70 gene was downregulated in the 10 μg/L dinotefuran group. It is known that HSP70 plays important roles in the folding and maturation of steroid hormone receptors (32). In conclusion, environmental concentrations of dinotefuran delayed the ecdysis activity of chironomids via affecting the transcriptions of key genes *ecr*, *usp*, *E74*, and *hsp70*.

### 4.2 Dinotefuran shifted sex ratios toward male by regulating related gene expressions

The endocrine-disrupting effect of neonicotinoids may not only affect the secretion of ecdysone but also induce a sex-dependent response. Our results observed that dinotefuran shifted sex ratios of adult midges toward male-dominated populations with increasing concentration. A previous study has reported that after chronic exposure to imidacloprid, clothianidin, and thiamethoxam, the sex of *C. dilutus* was affected with the proportion of males increasing (33). Sandrock et al. (34) observed that in laboratory experiments, chronic neonicotinoid exposure led to a male-biased offspring sex ratio in the solitary bee species *Osmia bicornis*. However, the molecular mechanisms affecting the sex ratio are not well understood. In this study, a preliminary analysis on the causes of sex ratio changes at the genetic level was made. Firstly, we did not observe significant changes in *err* gene expression. Gene *err*, as the estrogen-related receptor gene, together with estrogen receptor genes, is involved in the estrogen signaling pathway and induces transcriptional activation of estrogen response genes. In addition, it also participates in the regulation of glycolysis and plays an important role in metabolic regulation and growth and development. Even so, there was upregulation of *vtg* gene under 1 μg/L of dinotefuran. Vtg gene is considered to be a key biomarker in assessing the vertebrate and non-vertebrate estrogenic effects of pollutants (35). Our results indicated that environmental concentrations of dinotefuran may induce estrogen effect via regulating the expression of *vtg* gene. In addition, ecdysone-responsive genes play an important role in sexual differentiation. Therefore, sex-dependent changes could determine transcriptional processes (24). During the fourth larval stage, the gene expressions of *ecr*, *usp*, *E74*, and *vtg* exhibited a sex-dependent response with significant differences between males and females of Chironomidae. In this study, although we did not compare the differences in the expression of these genes (*ecr*, *usp*, *E74*, and *vtg*) between the sexes, the expression of these genes was dysregulated, suggesting that changes in the sex ratio may be associated with changes in these genes’ expressions. There have been relevant findings that neonicotinoids caused sex-related difference effects. For example, clothianidin exposure led to sex-related behavioral effects, including decreased motor activity and elevated anxiety-like behaviors, that were more pronounced in males. The concentrations of clothianidin, along with most metabolites in blood and urine, were higher in males than in females (36). These results manifested that neonicotinoids produced sex-related differential effects. However, there is a lack of research on the mechanism of sex ratio change. Our results provided a preliminary insight into the causes of the change in sex ratio.

## 5 Conclusion

In summary, the environmental concentration of dinotefuran delayed pupation and emergence via inhibiting ecdysis. Sex ratios of adults shifted toward male-dominated populations with dinotefuran increasing. The corresponding transcription of molting genes was significantly altered in the midge. The delayed development was closely related to energy disturbance and the regulation of ecdysone. The mechanism between energy interference and molting needs to be further explored. In conclusion, these results would improve our understanding of the potential endocrine-disrupting mechanisms of neonicotinoid insecticides to Chironomidae.

## Data Availability

All datasets generated for this study are included in the article/**Supplementary Material**. More detailed data are available on request to the corresponding author.
