# Supplementary material for: Long-term exposure to environmental concentration of dinotefuran disrupts ecdysis and sex ratio by dysregulating related gene expressions in Chironomus kiinensis
Source: Front Endocrinol (Lausanne). 2024 Sep 5;15:1459329. doi: 10.3389/fendo.2024.1459329 (PMC11410589; doi:10.3389/fendo.2024.1459329)
Supplement: Supplementary file 1 [file DataSheet1.docx]

**Supplemental Information**

**Long-term exposure to environmental concentration of dinotefuran disrupts ecdysis and sex ratio by** **dysregulating related gene expressions in *Chironomus kiinensis***

Fenghua Wei^a^, Shuangxin Wu^b,*^, Long Li^a^

^a^School of Chemistry and Environment, Jiaying University, Meizhou 514015, China

^b^School of Physics and Electrical Engineering, Jiaying University, Meizhou 514015, China

*Corresponding author

Shuangxin Wu

School of Physics and Electrical Engineering, Jiaying University, Meizhou 514015, China

E-mail: [409794313@qq.com](mailto:409794313@qq.com)

Submission to: ***Frontiers in Endocrinology***

**The file includes:**

Supporting Tables: 7

Supporting Figures: 0

References: Page S8

**Table S1.** Qualification parameters for analyzing dinotefuran, thiamethoxam-*d_3_* (internal standard), and imidacloprid-*d_4_* (surrogate standard) using HPLC-MS/MS.

| Compound | Transition mass (m/z) | Declustering Potential (V) | Collision energy (eV) | Retention time (min) |
| --- | --- | --- | --- | --- |
| Dinotefuran | 203.1→129.1^a a^ | 50 | 16 | 2.23 |
|  | 203.1→114.0^b^ | 50 | 17 |  |
| Imidacloprid-*d_4_* | 260.1→179.1^a^ | 100 | 30 | 1.51 |
|  | 260.1→213.1^b^ | 100 | 22 |  |
| Thiamethoxam-*d_3_* | 295.1→214.0^a^ | 120 | 18 | 1.20 |
|  | 295.1→131.9^b^ | 120 | 30 |  |

^a^ MS/MS transition used for quantification

^b^ MS/MS transition used for confirmation

**Table S2.** Measured concentration of dinotefuran in exposure solution at 5 and 10 d (Data are expressed as mean ± standard deviation (n = 3)).

| Group^a^ | Nominal concentration (μg/L) | Measured concentration (μg/L) | |
| --- | --- | --- | --- |
|  |  | 5 d | 10 d |
| Negative control | 0 | ND^a^ | ND |
| Solvent control (0.1% DMSO) | 0 | ND | ND |
| DIN_1 | 0.1 | 0.12±0.01 | 0.11±0.02 |
| DIN_2 | 1.0 | 1.21±0.23 | 1.08±0.19 |
| DIN_3 | 10.0 | 9.22±1.06 | 9.37±0.93 |

^a^ ND: Not detected.

**Table S3.** Time of different ecdysis stages in *C. kiinensis* after exposure to different concentrations of dinotefuran.

| Time of different ecdysis stages | NC^a^ | SC^b^ | 0.1 μg/L | 1 μg/L | 10 μg/L |
| --- | --- | --- | --- | --- | --- |
| The time of the last larva (days) | 16 | 15 | 16 | 19 | 17 |
| First pupae time (days) | 10 | 11 | 10 | 13 | 13 |
| Time for the last pupa (days) | 17 | 16 | 17 | 20 | 19 |
| First emergence time (days) | 11 | 12 | 11 | 14 | 14 |
| Last emergence time (days) | 18 | 18 | 18 | 21 | 20 |

^a^NC: Negative control;

^b^SC: Solvent control.

**Table S4.** Cumulative emergence rates of *C. kiinensis* after exposure to different concentrations of dinotefuran.

| Days (d) | Cumulative emergence rate (%) | | | | |
| --- | --- | --- | --- | --- | --- |
|  | NC^a^ | SC^b^ | 0.1 μg/L | 1 μg/L | 10 μg/L |
| 10 | 0 | 0 | 0 | 0 | 0 |
| 11 | 5 | 0 | 5 | 0 | 0 |
| 12 | 10 | 10 | 5 | 0 | 0 |
| 13 | 20 | 15 | 10 | 0 | 0 |
| 14 | 35 | 35 | 25 | 15 | 10 |
| 15 | 55 | 50 | 40 | 20 | 15 |
| 16 | 65 | 70 | 60 | 35 | 20 |
| 17 | 80 | 85 | 70 | 40 | 20 |
| 18 | 90 | 90 | 80 | 45 | 25 |
| 19 | 90 | 90 | 80 | 50 | 25 |
| 20 | 90 | 90 | 80 | 50 | 30 |
| 21 | 90 | 90 | 80 | 55 | 30 |

^a^NC: Negative control;

^b^SC: Solvent control.

**Table S5.** The number of survival adult male and female in *C. kiinensis* along with the proportion of each sex after exposure to dinotefuran.

| Group | Male | Female | Male (%) | Female (%) |
| --- | --- | --- | --- | --- |
| NC_1^a^ | 11 | 8 | 57.9 | 42.1 |
| NC_2 | 8 | 9 | 47.1 | 52.9 |
| NC_3 | 8 | 10 | 44.4 | 55.6 |
| SC_1^b^ | 10 | 8 | 55.6 | 44.4 |
| SC_2 | 10 | 9 | 52.6 | 47.4 |
| SC_3 | 8 | 9 | 47.1 | 52.9 |
| DIN_1_1 | 10 | 7 | 58.8 | 41.2 |
| DIN_1_2 | 8 | 8 | 50.0 | 50.0 |
| DIN_1_3 | 6 | 8 | 42.9 | 57.1 |
| DIN_2_1 | 8 | 5 | 61.5 | 38.5 |
| DIN_2_2 | 7 | 3 | 70.0 | 30.0 |
| DIN_2_3 | 6 | 4 | 60.0 | 40.0 |
| DIN_3_1 | 5 | 2 | 71.4 | 28.6 |
| DIN_3_2 | 3 | 2 | 60.0 | 40.0 |
| DIN_3_3 | 4 | 2 | 66.7 | 33.3 |

^a^NC: Negative control;

^b^SC: Solvent control.

**Table S6.** Adenosine triphosphate (ATP) level in the larva of *C. kiinensis* after 11 days of exposure to different concentrations of dinotefuran.

| Exposure group | ATP level (μmol/g protein)/(n=3) | | |
| --- | --- | --- | --- |
| Negative control | 2.23 | 2.13 | 1.73 |
| Solvent control | 2.16 | 2.06 | 1.75 |
| 0.1 μg/L | 1.34 | 1.94 | 2.04 |
| 1 μg/L | 1.29 | 1.40 | 1.58 |
| 10 μg/L | 1.29 | 1.68 | 1.01 |

**Table S7.** Gene expressions related to ecdysone and estrogen effect in larva after 11 days of exposure to dinotefuran.

| Gene | Relative gene expression | | | | |
| --- | --- | --- | --- | --- | --- |
|  | Negative control | Solvent control | 0.1 μg/L | 1 μg/L | 10 μg/L |
| *ecr* (n=3) | 0.82 | 1.00 | 0.81 | 0.66 | 0.45 |
|  | 1.12 | 1.19 | 0.98 | 0.75 | 0.72 |
|  | 0.99 | 0.81 | 1.12 | 0.72 | 0.51 |
| *usp (n=3)* | 0.99 | 0.95 | 0.92 | 0.56 | 0.78 |
|  | 1.24 | 1.11 | 1.2 | 0.83 | 0.92 |
|  | 0.87 | 0.93 | 0.91 | 0.67 | 0.64 |
| *E74* (n=3) | 1.16 | 0.93 | 0.53 | 0.51 | 0.75 |
|  | 0.85 | 1.12 | 0.78 | 0.50 | 0.72 |
|  | 1.01 | 0.94 | 0.79 | 0.82 | 0.51 |
| *hsp70* (n=3) | 0.97 | 1.09 | 0.91 | 1.15 | 0.85 |
|  | 1.04 | 1.10 | 0.98 | 0.95 | 0.72 |
|  | 0.93 | 0.81 | 0.74 | 1.06 | 0.61 |
| *err* (n=3) | 0.91 | 1.00 | 0.83 | 1.11 | 1.45 |
|  | 1.21 | 1.08 | 0.55 | 0.81 | 1.06 |
|  | 0.90 | 0.93 | 0.79 | 1.22 | 1.43 |
| *vtg* (n=3) | 1.07 | 1.00 | 0.93 | 1.33 | 1.57 |
|  | 1.05 | 1.19 | 0.98 | 1.75 | 0.95 |
|  | 0.93 | 0.81 | 0.83 | 1.87 | 1.38 |
